# Bifunctional Poly(ionic liquid) Membranes for CO_2_ Utilization

**DOI:** 10.3390/polym18091129

**Published:** 2026-05-03

**Authors:** Maria Atlaskina, Kirill Smorodin, Sergey Kryuchkov, Artem Atlaskin, Nikolay Lukashov, Anton Petukhov, Andrey Vorotyntsev, Ilya Vorotyntsev

**Affiliations:** 1 Laboratory of SMART Polymeric Materials and Technologies, Mendeleev University of Chemical Technology of Russia, 125047 Moscow, Russia; 2 Chemical Engineering Laboratory, Lobachevsky State University of Nizhny Novgorod, 603022 Nizhny Nogorod, Russia

**Keywords:** polymeric ionic liquids, gas separation membranes, CO_2_ capture, CO_2_ cycloaddition, bifunctional materials, carbon capture and utilization (CCU)

## Abstract

In this study, the task of integrating capture and conversion of CO_2_ into a single material platform is realized by developing bifunctional membranes based on polymer ionic liquids (PILs). The novelty of this work lies in the fabrication and comprehensive evaluation of PIL-based membrane materials that combine catalytic activity toward CO_2_ conversion with gas separation performance within one material system. In contrast to most previously reported imidazolium-based PILs, which have mainly been considered either as catalysts or as membrane materials, the present approach focuses on their dual functionality under both catalytic and gas transport conditions. A series of imidazolium-based PILs, including homopolymers and block copolymers with polystyrene, were synthesized. The materials were characterized to determine their catalytic activity during the cycloaddition of CO_2_ to epichlorohydrin and to determine their gas transport properties using pure gases (N_2_, O_2_, CO_2_) and a simulated dry flue gas mixture; membrane morphology was studied by scanning electron microscopy. Block copolymers exhibited higher catalytic conversions (up to 82.7%) than homopolymers, with selectivities above 93%. Chloride-containing block copolymers gave the best combination of CO_2_ permeability (up to 7.5 Barrer) and CO_2_/N_2_ selectivity (18–22) under mixed-gas conditions. Iodide-containing analogs demonstrated higher selectivity (up to 30) but lower CO_2_ permeability. Morphological analysis confirmed the presence of dense, defect-free structures in materials with the chloride anion, while materials with the iodide anion showed increased free volume and microheterogeneity. These results indicate that by altering the polymer and anion architecture, PIL-based membranes can effectively combine catalytic activity with selective CO_2_ transport, providing a promising avenue for enhancing carbon capture and utilization processes.

## 1. Introduction

The rapid increase in anthropogenic carbon dioxide (CO_2_) emissions remains one of the major global environmental challenges of the twenty-first century. Recent assessments indicate that global CO_2_ emissions from fossil fuel combustion and industrial processes exceed 36–37 Gt annually, with large stationary sources such as power plants and metallurgical facilities accounting for a significant fraction of these emissions [1]. Decreasing CO_2_ emissions from such sources requires the deployment of technologies capable of efficiently capturing and processing large volumes of carbon dioxide from gas streams.

For several decades, efforts in this field have primarily focused on the concept of Carbon Capture and Storage (CCS), in which CO_2_ is separated from industrial gas streams and permanently stored in geological formations [2,3]. Although CCS remains a technically feasible strategy, its widespread implementation is often limited by high capital costs and infrastructure requirements. In recent years, increasing attention has been directed toward Carbon Capture and Utilization (CCU) strategies, where captured CO_2_ is converted into valuable chemicals materials [4]. By transforming CO_2_ from a waste product into a carbon feedstock, CCU technologies offer a pathway toward both emission reduction and sustainable chemical production.

Among the various chemical transformations of CO_2_, the cycloaddition of CO_2_ to epoxides to form cyclic carbonates has attracted considerable interest [5,6,7]. This reaction proceeds with theoretical atom economy of 100%, avoids the formation of stoichiometric byproducts, and enables the direct incorporation of CO_2_ into industrially relevant products. Cyclic carbonates are widely used as polar aprotic solvents, intermediates in organic synthesis, electrolytes in lithium-ion batteries, and precursors for the production of polycarbonates and polyurethanes. As a result, extensive research efforts have been devoted to the development of efficient catalysts for this transformation, including metal complexes, heterogeneous catalysts, and ionic liquid-based catalytic systems.

Despite significant progress in catalyst design, the large-scale implementation of CO_2_ conversion technologies remains limited by the problem of upstream CO_2_ capture and purification. Industrial flue gases typically contain only 5–15 vol.% CO_2_, with nitrogen being the dominant component, along with oxygen, water vapor, and trace impurities such as SO_x_ and NO_x_. Therefore, CO_2_ must first be separated and concentrated from these gas streams before catalytic conversion. Common capture technologies, including chemical absorption using amine solutions [8,9,10,11], adsorption on porous materials [12,13], cryogenic separation [14,15], membrane gas separation [16,17,18,19,20], and hybrid gas separation methods [21,22,23], are typically implemented as process units independent from the catalytic reactor. Such process configurations necessarily require intermediate gas compression, heating and cooling cycles, and transport of purified CO_2_ streams, resulting in significant energy losses and increased capital costs.

Overcoming these limitations requires the development of integrated technologies capable of combining separation and conversion processes within a single process unit. This concept is closely related to the process intensification approach, which aims to improve process efficiency by integrating multiple functions into compact, highly efficient devices. In the context of CO_2_ utilization, one promising approach is the development of multifunctional materials capable of simultaneously performing selective CO_2_ capture and catalytic conversion. Bifunctional membranes are an excellent candidate for such materials, as they could enable the development of reactors that capture CO_2_ from a gas mixture using a selective membrane and immediately convert it into valuable products. This would exclude intermediate separation steps and reduce both energy consumption and process complexity.

Polymeric membranes represent one of the most attractive platforms for CO_2_ separation due to their low energy requirements, operational simplicity, and modular scalability. In recent years, important research has been devoted to improving membrane performance for CO_2_/N_2_ separation, which is particularly relevant for flue gas treatment. Polymeric membranes generally exhibit a well-known trade-off between permeability and selectivity, commonly described by the Robeson plot [24]. Although significant progress has been made in developing advanced membrane materials that can approach or exceed this upper bound, further improvements often require new classes of "smart" polymers with specific interactions with CO_2_ molecules.

In this context, polymeric ionic liquids (PILs) are a promising class of functional materials for gas separation and catalysis [25,26,27]. PILs combine the unique properties of ionic liquids—chemical affinity for CO_2_, low vapor pressure, and a tunable chemical structure—with the processability of polymeric materials [28]. The presence of ionic functional groups in the polymer backbone or side chains can significantly enhance CO_2_ sorption and facilitate selective transport through the membrane matrix. Numerous studies have shown that PIL-based membranes exhibit favorable CO_2_/N_2_ separation properties due to specific interactions between the quadrupole CO_2_ molecule and ionic centers within the polymer [29,30,31]. Among these materials, vinylimidazolium-based PILs have been identified as especially promising CO_2_-philic membrane materials. Carlisle et al. showed that the separation performance of poly(vinylimidazolium) membranes strongly depends on the chemical structure of the ionic moieties and can be significantly improved by forming PIL/ionic liquid composite films [32]. Li et al. further demonstrated that such imidazolium-based PIL and PIL-RTIL membranes are effective for CO_2_/N_2_ separation relevant to flue-gas purification, with the best systems exhibiting performances close to the Robeson upper bound [33]. Later, Nellepalli et al. extended this concept to imidazolium-based copoly(ionic liquid) membranes and showed that variation of the copolymer structure provides an additional tool for tuning membrane preparation and CO_2_/N_2_ separation performance [34]. These studies clearly established vinylimidazolium-based homo- and copoly(ionic liquid)s as attractive membrane materials for selective CO_2_ transport.

At the same time, imidazolium-based ionic liquids are known as efficient catalysts for the cycloaddition of CO_2_ to epoxides [35,36,37,38]. In these systems, catalytic activity is typically associated with nucleophilic anions (such as halides) that promote epoxide ring opening and facilitate the formation of CO_2_ to form cyclic carbonates. Inclusion of such catalytic ionic fragments into polymer structures provides an attractive strategy for developing materials that combine catalytic functionality with structural stability. Rational molecular design of imidazolium-containing polymeric ionic liquids may allow the creation of materials capable of both selective transfer of CO_2_ and catalytic transformation. Nevertheless, although vinylimidazolium-based PILs and related copoly(ionic liquid)s have been extensively investigated as gas-separation media, their integration with catalytic CO_2_ conversion has remained largely unexplored. This creates a clear rationale for developing bifunctional vinylimidazole-based materials and styrene copolymers that can both separate CO_2_ from gas mixtures and catalyze its conversion into value-added cyclic carbonates.

This article presents the results of the development and characterization of new bifunctional materials based on polymeric ionic liquids containing imidazolium groups for CO_2_ capture and catalytic conversion. PILs containing imidazolium groups were synthesized, and flat gas separation membranes based on them were fabricated. The catalytic activity of the PILs was studied in a model of cycloaddition reaction of CO_2_ with epichlorohydrin. The gas separation efficiency of the membranes was evaluated using pure gases (nitrogen, oxygen, carbon dioxide), and a three-component gas mixture based on them, characteristic of the composition of industrial flue gases. The results obtained in this study give new insights into the correlation between polymer structure, gas transport properties, and catalytic activity. More broadly, this work demonstrates the potential of multifunctional polymeric materials as a basis for the development of compact and energy-efficient technologies for the integrated capture and use of CO_2_.

## 2. Materials and Methods

### 2.1. Materials and PIL Preparation

The following materials were used: 1-Vinylimidazole (VIm, 99%) was purchased from Macklin Inc. (Shanghai, China); styrene (Sty, ≥99%) and potassium iodide (KI, >99.8%) were obtained from Rushim (Moscow, Russia); azobisisobutyronitrile (AIBN, >98%) was purchased from Clearsynth (Mumbai, India); 2-Chloroethan-1-ol (99%) was obtained from Acros Organics (Geel, Belgium); chloroacetic acid (MCA, ≥99%) was purchased from Sigma-Aldrich (Taufkirchen, Germany); dimethylformamide (DMF, 99%), N-methylpyrrolidone (NMP, >99%), and hexane (99%) were supplied by LLC "Component-Reactive" (Moscow, Russia); dimethylacetamide (DMA, >99%) was obtained from JSC VECTON (Saint Petersburg, Russia); tetrahydrofuran (THF, >99%) was purchased from JSC "ECOS-1" (Moscow, Russia); chloroform (>99%), methanol (MeOH, >99%), toluene (PhMe, 99%), and acetonitrile (ACN, 99%) were supplied by Chimmed (Moscow, Russia); and epichlorohydrin (ECH, >99%) was purchased from Chemical Line Co., Ltd. (Saint Petersburg, Russia).

The gas mass properties of the membranes were determined using the gases nitrogen (≥99.999%), carbon dioxide (≥99.99%), and oxygen (≥99.999%), which were purchased from Monitoring LLC, Russia; LLC “NII KM” (Moscow, Russia); and LLC “Voessen” (Moscow, Russia). Pure helium (≥99.9999%) and argon (≥99.9999%) purchased from LLC “NII KM” (Moscow, Russia) were used for analytical purposes.

The chemical structures of the synthesized polymeric ionic liquids were confirmed by ^1^H NMR spectroscopy using an Ascend400 NMR spectrometer (Bruker, Zurich, Switzerland) with D_2_O, CDCl_3_, and DMSO-d_6_ as deuterated solvents. Additional structural characterization was carried out by FTIR spectroscopy on a Bruker Equinox 55/s spectrometer (Ettlingen, Germany) in the range of 4000–550 cm^−1^ to identify the characteristic functional groups of the synthesized materials.

The thermal transitions of the synthesized polymeric ionic compounds were studied using differential scanning calorimetry (DSC) on a Phoenix F1 calorimeter (Netzsch, Selb, Germany). Measurements were performed under an inert atmosphere to prevent oxidative degradation and ensure reproducibility of the thermal effects. To eliminate the temperature history of the samples, a standard heating–cooling–heating regime was used. In the first step, the samples were heated to 300 °C at a rate of 10 °C min^−1^, which ensured structural relaxation and eliminated the effects of previous processing. They were then cooled to 0 °C, followed by a second heating step at the same rate (10 °C min^−1^), the data from which were used to analyze the thermal transitions. The glass transition temperature (Tg) was determined from the inflection point of the baseline on the heat flow curve. The melting temperature (Tm) was determined as the temperature of the maximum of the corresponding endothermic peak.

**Figure 1:**
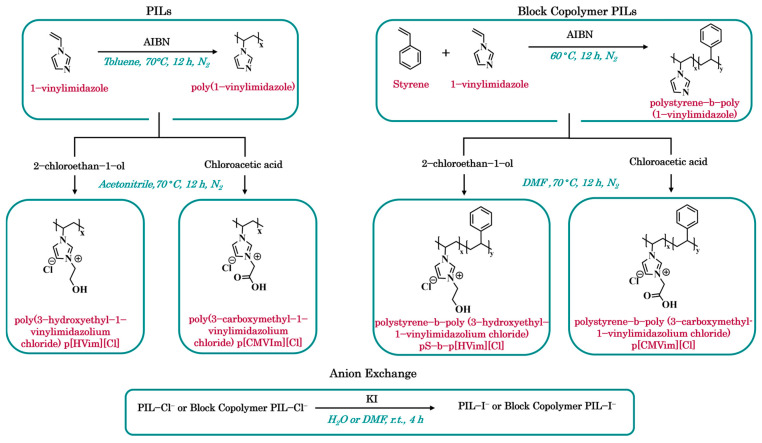
PIL synthesis scheme.

Synthesis of PILs. Poly(1-vinylimidazole) (p[VIm]) was obtained via radical polymerization of 1-vinylimidazole (2 wt% AIBN, toluene, 70 °C, 12 h, N_2_). The product was isolated by filtration and vacuum-dried (70 °C, 12 h), yielding 71% of a white powder. For quaternization, p[VIm] was reacted with either 2-chloroethan-1-ol or chloroacetic acid (2:1 molar ratio) in acetonitrile (70 °C, 12 h, N_2_). The resulting poly(3-hydroxyethyl-1-vinylimidazolium chloride) (p[HVIm][Cl]) and poly(3-carboxymethyl-1-vinylimidazolium chloride) (p[CMVIm][Cl]) were filtered and vacuum-dried (60 °C, 24 h), giving light-yellow powders in 76% and 74% yields, respectively (Figure 1).

[HVIm][Cl]: ^1^HNMR (300MHz, deuteriumoxide) δ 7.30–7.24 (m, 2H), 6.82–6.49 (m, 1H), 4.20 (s, 2H), 3.99–3.54 (m, 2H), 2.13 (d, 6H).

[CMVIm][Cl]: ^1^H NMR (300 MHz, deuterium oxide) δ 2.02 (d, 6H), 7.36 (d, 1H), 7.44 (d, 1H), 8.70 (s, 1H), 4.74 (s, 2H), 4.28–4.06 (m, 1H).

Synthesis of Block Copolymer PILs. The block copolymer pS-b-p[VIm] was first prepared by copolymerizing styrene and 1-vinylimidazole (1:1 mol ratio, 2 wt% AIBN, 60 °C, 12 h, N_2_). The product was precipitated in cold diethyl ether/hexane (20/80 *v*/*v*), filtered, washed with cold hexane, and vacuum-dried (80 °C, 24 h) to afford a white powder (53% yield). Quaternization with 2-chloroethan-1-ol or chloroacetic acid (2:1 molar ratio) was carried out in DMF (70 °C, 12 h, N_2_). The obtained pS-b-p[HVIm][Cl] and pS-b-p[CMVIm][Cl] were isolated by filtration and vacuum-dried (60 °C, 24 h) to obtain light-yellow powders in 71% and 70% yields, respectively (Figure 1).

pS-b-p[HVIm][Cl]: ^1^H NMR (300 MHz, chloroform-d) δ 7.29 ppm (s, 1H), 7.27–6.86 ppm (br.s, 2H), 6.80–6.51 ppm (br.s, 4H), 6.49 ppm (s, 1H), 3.88 ppm (t, 2H), 3.79 ppm (m, 1H), 3.68 ppm (t, 2H), 3.08 ppm (m, 1H), 2.10–1.16 ppm (m, 7H), 0.91 ppm (m, 3H).

pS-b-p[CMVIm][Cl]: ^1^H NMR (300 MHz, DMSO-d6) δ 6.80 (dd, 2H), 4.52–4.48 (m, 2H), 4.46–4.37 (m, 3H), 4.27 (s, 1H), 4.07–3.80 (m, 1H), 3.63–3.54 (m, 1H), 2.54–2.48 (m, 4H), 2.00–1.07 (m, 6H).

Anion Exchange. To replace chloride anions with iodide, an aqueous solution containing a 10-fold molar excess of KI was added dropwise under stirring to an aqueous solution of the PILs or to a solution of the block copolymer PILs in dry DMF (5 wt.%). The reactions were carried out at room temperature for 4 h. After completion, a large excess of diethyl ether or acetone was added to precipitate the iodide polymer. The precipitate was collected by filtration, washed with diethyl ether followed by a diethyl ether/acetone mixture (70/30 *v*/*v*), and dried in a vacuum oven at 35 °C to a constant weight (approximately 40 h). Successful anion exchange was confirmed qualitatively by AgNO_3_ testing and aqueous ammonia treatment, which showed the characteristic behavior of AgCl and AgI, respectively.

pS-b-p[HVIm][I]: ^1^H NMR (300 MHz, DMSO-d6) δ 7.10–7.20 ppm (s, 1H), 7.20–6.80 ppm (br.s, 2H), 6.78–6.48 ppm (br.s, 4H), 6.41–6.44 ppm (s, 1H), 3.88 ppm (t, 2H), 3.77 ppm (m, 1H), 3.58–3.64 ppm (t, 2H), 3.03–3.07 ppm (m, 1H), 2.08–1.14 ppm (m, 7H), 0.89–0.92 ppm (m, 3H).

pS-b-p[CMVIm][I]: ^1^H NMR (300 MHz, DMSO-d6) δ 6.74–6.79 ppm (dd, 2H), 4.46–4.42 ppm (m, 2H), 4.40–4.31 ppm (m, 3H), 4.22–4.25 ppm (s, 1H), 4.03–3.78 ppm (m, 1H), 3.60–3.50 ppm (m, 1H), 2.52–2.46 ppm (m, 4H), 1.99–1.06 ppm (m, 6H).

### 2.2. Catalytic Activity

The catalytic activity of the polymeric ionic liquids (PILs) was evaluated during the cycloaddition reaction of CO_2_ to the epoxide epichlorohydrin. The reaction was carried out in a reactor at 90 °C for 4 h with a catalyst loading of 2 mol%. A detailed description of the setup and experimental procedure is provided in [25]. The qualitative composition of the reaction mixture was determined using a chromatograph–mass spectrometer, while the quantitative analysis was performed on a gas chromatograph equipped with a flame ionization detector. In both cases, high-purity helium (≥99.99999 vol%) was used as the carrier gas with a flow rate of 1 mL min^−1^. A flow split of 1:10 was used for GC-FID, and 1:20 for GC-MS. The analysis was performed on TR-5MS capillary columns with a stationary phase consisting of 5% diphenylpolysiloxane and 95% dimethylpolysiloxane. For GC-FID, the column temperature was held at 50 °C for 6 min, then heated to 250 °C at a rate of 10 °C/min and held for 2 min. For GC-MS, the temperature program included heating to 80 °C for 6 min, then heating to 310 °C at a rate of 10 °C min^−1^, followed by holding for 7 min. The injector temperature in both cases was 250 °C. Ionization in the mass spectrometer was carried out through electron impact with an energy of 70 eV.

### 2.3. Polymer Film Formation by Dry Forming

To produce films by dry forming (solution casting), a suitable solvent was selected for each polymeric ionic liquid to ensure complete polymer dissolution and an optimal evaporation rate. Dimethylformamide (DMF), dimethylacetamide (DMA), tetrahydrofuran (THF), N-methylpyrrolidone (NMP), chloroform, water (H_2_O), ethanol (EtOH), and methanol (MeOH) were tested. The prepared homogeneous polymer solution, with a concentration of 15–20 wt.%, was filtered using a syringe filter to remove insoluble particles.

A glass or fluoroplastic plate was used as a substrate; it was thoroughly cleaned and degreased. Using a film knife-applicator with a fixed gap, the solution was uniformly applied to the surface, forming a layer of the desired thickness.

To prevent defects caused by rapid evaporation, the substrate with the applied solution was placed under a hood, which ensured slow and controlled solvent removal at room temperature. After complete drying, the formed film was carefully separated from the substrate and dried in a vacuum oven at 40 °C until a constant weight was achieved.

### 2.4. Gas Transport Properties of Membranes

The membrane performance was first evaluated by measuring the single-gas permeance of nitrogen, oxygen, and carbon dioxide. To assess the effect of pressure on the gas-transport properties of the prepared membranes, single-gas permeance measurements were carried out at two pressures, 0.1 and 0.5 MPa. Subsequently, mixed-gas permeance tests were performed using a ternary feed mixture containing 82.0 mol.% nitrogen, 5.0 mol.% oxygen, and 13.0 mol.% carbon dioxide, simulating dry flue gas. In this case, the membranes were tested at a feed pressure of 0.15 MPa. All experiments were performed at 25 °C.

**Figure 2:**
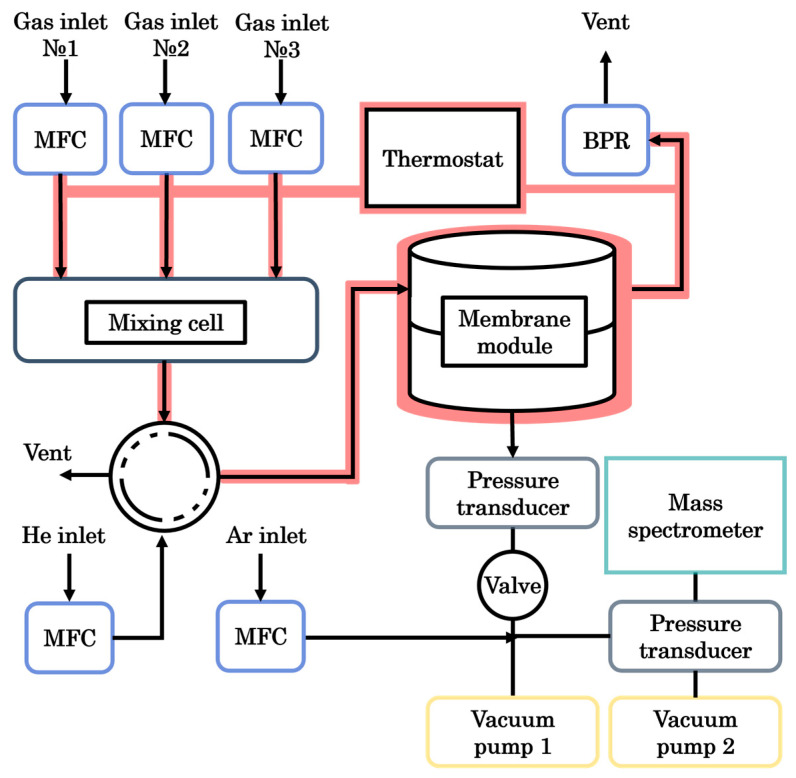
Scheme of the experimental setup for evaluating the gas transport properties of membranes.

All experiments were performed on a custom-built permeation setup integrated with a quadrupole mass spectrometer for real-time gas analysis (Figure 2). Gas supply was precisely controlled using mass flow controllers (FG-201 CV, Bronkhorst, Veenendaal, NW, The Netherlands), which could be operated independently or in mixing mode to generate the desired gas composition directly into a mixing cell. Additional flow controllers (F-201 CV and F-201 CM, Bronkhorst, Veenendaal, NW, The Netherlands) delivered helium for system purging and argon as an internal standard, provided that the permeance of these gases was not under investigation. A pneumatically actuated two-position valve directed the feed from either the mixing cell or the helium line to the membrane module. On the retentate side, a pressure regulator (P702 CM, Bronkhorst, Veenendaal, NW, The Netherlands) maintained constant pressure across the membrane.

Gas permeating through the membrane entered a vacuum chamber connected to a pumping station (Hi-Cube ECO 300, Pfeiffer, Asslar, Germany). Permeate-side pressure was monitored with a pressure transducer (MPT200, Pfeiffer, Asslar, Germany). To protect the vacuum system in the event of membrane failure, a solenoid-operated diaphragm valve (DVC 025 PX, Pfeiffer, Asslar, Germany) was installed between the module and the pumps. From there, the permeate stream was directed into a quadrupole mass spectrometer (QMG 250 M2, Pfeiffer, Asslar, Germany) backed by a separate pumping unit (Hi-Cube 80, Pfeiffer, Asslar, Germany). A second pressure transmitter monitored the vacuum level inside the spectrometer chamber.

Prior to each measurement, the membrane module was flushed with helium at a flow rate of 50–150 cm^3^·min^−1^ to remove any residual gases from previous runs. Simultaneously, the mixing tank was charged with either pure gases or a pre-mixed blend at a total flow rate of up to 750 cm^3^·min^−1^. Argon was continuously introduced into the vacuum-side lines at 4 cm^3^·min^−1^, except during argon permeance tests. The switching valve connecting the mixing tank to the feed side was actuated with an 8 ms delay, ensuring a controlled transition. Permeation data were acquired by the mass spectrometer with a refresh rate of 1 ms, while pressure readings and mass flow signals were recorded using FlowPlot V.3.35 software. Mass spectra and permeate-side pressure were captured via PV MassSpec V.21.04.01-b and PV TurboViewer V.01.04.00. This configuration enabled continuous, real-time data acquisition, allowing permeance values to be calculated at any given point using standard equations.

The permeability coefficient P is determined by the formula

(1) P=Ji·L∆p·A,

where $J_{i}$ is the volumetric flow rate of component i, (cm^3^(STP)∙s^−1^); *L* is the membrane thickness (cm); ∆p is the partial pressure difference in component *i* across the membrane (cmHg); and A is the membrane area (cm^2^).

The selectivity of the membrane is calculated according to the equation

(2) α=PAPB,

where $P_{A}$ is the permeability of component A, and $P_{B}$ is the permeability of component B.

The diffusion coefficient (D) was obtained through the time-lag (θ) technique [39]:

(3) D=l26·θ

The diffusion coefficient was determined using the transition region of the permeability kinetic curve. The sorption coefficient (S) was calculated using the formula

(4) S=PD

The mass spectrometer software converts the signal intensity of each component into its corresponding partial pressure. On this basis, the volumetric flow rate of the permeate can be estimated using the following equation:

(5) JiJAr=pipAr,

where $J_{Ar}$ is the volumetric flow rate of argon, cm^3^ min^−1^; pi is the partial pressure of component i in the permeate, cmHg; and $p_{Ar}$ is the partial pressure of argon in the permeate, cmHg.

To quantify the flux of each gas component through the membrane, a calibration procedure was carried out following the methodology described by Fraga et al. [40]. For mass spectrometric analysis, the characteristic *m*/*z* signals selected for the gas mixture components were 28 for N_2_, 32 for O_2_, and 44 for CO_2_.

The CO_2_ flux at *m*/*z* = 44 was calculated using Equation (5) with correction for the relative detector sensitivity [40]. The detector signals for oxygen (*m*/*z* = 32) and nitrogen (*m*/*z* = 28) were additionally affected by fragment contributions arising from CO_2_, including the signal at *m*/*z* = 28. Therefore, the individual contribution of each component to the total detector response was evaluated in a preliminary calibration study using a binary CO_2_/N_2_ mixture generated by dynamic flow mixing, and the corresponding correction factors were subsequently applied. The measurement error did not exceed ±2.2% of the recorded value. The mass transfer properties of each PIL membrane were measured three to five times. The values were averaged, and the sample-to-sample deviation did not exceed 2.9%.

## 3. Results and Discussion

**Figure 3:**
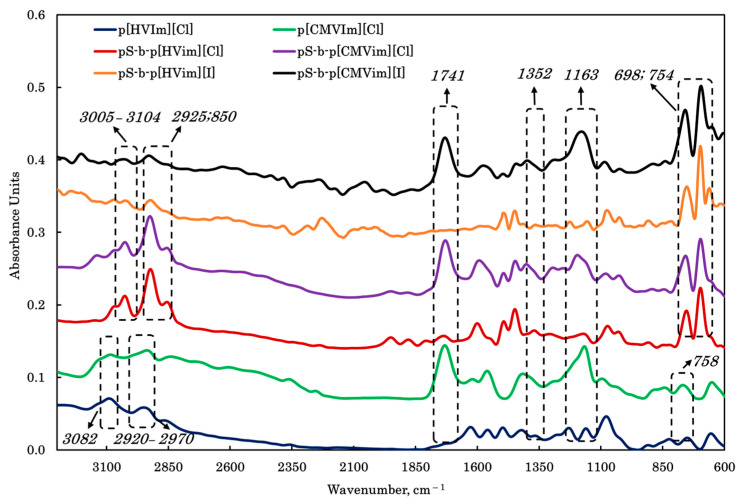
FTIR spectra of polymeric ionic liquids.

The FTIR spectra of the synthesized polymeric ionic liquids are presented in Figure 3. All samples showed a characteristic band at 1163 cm^−1^, assigned to the in-plane C–H deformation of the imidazole ring [41]. For the PILs containing chloroacetic acid fragments, the absorption at 1741 cm^−1^ confirmed the presence of the carbonyl group, indicating successful functionalization of the imidazole unit. In p[HVIm][Cl], pS-b-p[HVIm][Cl] and pS-b-p[HVIm][I], the band at 1352 cm^−1^ was attributed to C–OH bending vibrations, supporting the incorporation of the hydroxyethyl substituent [42]. Styrene-containing samples exhibited the expected aromatic C–H out-of-plane vibrations at 754 and 698 cm^−1^, as well as C–H stretching bands in the 3005–3104 cm^−1^ region [43]. The absorptions at 2925–2850 cm^−1^ were associated with aliphatic C–H stretching in the polymer backbone, while the band near 3082 cm^−1^ corresponded to C–H vibrations of the imidazole ring. Additional bands at 2946 cm^−1^ for p[HVIm][Cl] and 2916 cm^−1^ for p[CMVIm][Cl] were assigned to methylene C–H stretching in both the main chain and the side substituents [44]. The band around 758 cm^−1^ was also characteristic of out-of-plane C–H deformation in the imidazole ring.

**Figure 4:**
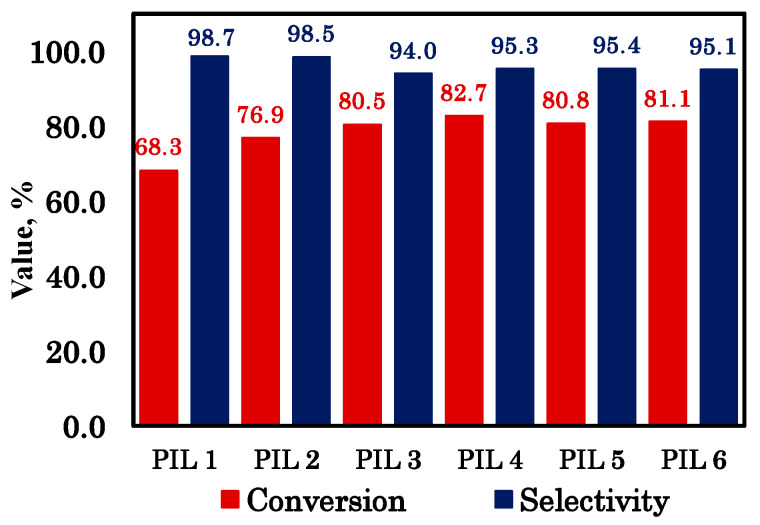
Catalytic characteristics of the PILs.

The catalytic characteristics of the PILs (epichlorohydrin conversion) are shown in Figure 4. The values for PIL 1–4 were published elsewhere [25]. For clarity, the PILs are numbered here and throughout this work: PIL 1—p[CMVim][Cl], PIL2—p[HVim][Cl], PIL 3—pS-b-p[CMVim][Cl], PIL 4—pS-b-p[HVim][Cl], PIL 5—pS-b-p[CMVim][I], PIL 6—pS-b-p[HVim][I].

The analysis of the catalytic activity of the PILs during the cycloaddition of CO_2_ to epichlorohydrin reveals clear structure–activity trends. Homopolymers without a polystyrene block (PIL 1 and PIL 2) give lower conversions of 68.3% and 76.88%, respectively, whereas all block copolymers (PIL 3–PIL 6) show significantly higher conversions in the range of 80.53 –82.69%, confirming that the inclusion of the polystyrene segment can increase the conversion to almost 15%. The higher conversions observed for the block copolymers are most likely associated with the effect of polymer architecture: the introduction of the polystyrene block leads to a microphase-separated nanostructure, which may create a more favorable local environment around the ionic catalytic sites and improve the accessibility of the reactants to these centers. Among the block copolymers, the HVIm-based materials (PIL 4 and PIL 6) exhibit slightly higher conversions than their CMVIm analogs—82.69% vs. 80.53% for the chloride series and 81.14% vs. 80.77% for the iodide series—indicating a modest cation effect in favor of HVIm. The slightly higher conversions of the HVIm-based materials may be attributed to the hydroxyethyl substituent, which likely provides a more favorable balance between hydrogen-bond-assisted epoxide activation and preservation of halide accessibility than the carboxymethyl group under the selected reaction conditions. Regarding the anion, chloride-containing PIL 3 and PIL 4 give conversions of 80.53% and 82.69%, respectively, while the corresponding iodide derivatives PIL 5 and PIL 6 afford 80.77% and 81.14%. The differences are small, suggesting that under the reaction conditions the anion effect is less pronounced than the influence of the polymer architecture. In terms of selectivity, the homopolymers show the highest values (>98%), whereas the block copolymers exhibit slightly lower selectivities (93.95–95.43%), with the chloride-based block copolymers giving selectivities of 93.95% (PIL 3) and 95.27% (PIL 4) and the iodide-based ones affording 95.43% (PIL 5) and 95.08% (PIL 6). Overall, the block copolymer with the HVIm cation and chloride anion—pS-b-p[HVIm][Cl] (PIL 4)—provides the best balance, with a conversion of 82.69% and a selectivity of 95.27%, making it the most efficient catalyst among the studied materials. The main reaction product identified by GC–MS was chloropropylene carbonate. Minor byproducts included 2,3-dichloropropan-1-ol and trace amounts of vinyl chloroacetate and 1-chloro-3-iodopropan-2-ol. The very high selectivity observed for all PILs indicates that the dominant reaction pathway is halide-assisted epoxide ring opening followed by CO_2_ insertion and intramolecular cyclization, whereas the byproducts most likely arise from incomplete turnover of the ring-opened intermediate or from minor side transformations. The slightly lower selectivity of the block copolymers compared with the homopolymers suggests that their more open and microphase-separated structure facilitates conversion, but at the same time slightly increases the probability of such secondary pathways.

A supposed mechanism for the cycloaddition of CO_2_ to epichlorohydrin over the synthesized PILs involves cooperative activation of the epoxide by the ionic pair. The imidazolium fragment, together with the pendant hydroxyethyl or carboxymethyl group, promotes epoxide polarization through electrophilic and hydrogen-bonding interactions, while the halide anion acts as the nucleophile responsible for ring opening. The resulting alkoxide intermediate then reacts with CO_2_ to form an alkylcarbonate species, followed by intramolecular cyclization to the cyclic carbonate with regeneration of the halide anion. Such an interpretation is consistent with the generally accepted mechanism of organocatalysed CO_2_–epoxide cycloaddition and with reports showing that hydroxy-functionalized imidazolium catalysts facilitate epoxide activation through protic hydrogen-bonding interactions [45,46]. In our previous study [25], the higher activity of the hydroxyethyl-functionalized PILs was associated with the moderate hydrogen-bonding ability of the hydroxyl group, whereas the improved performance of the styrene-containing block copolymers was attributed to a micellar catalytic effect that increases the local concentration of CO_2_ and epoxide near the ionic catalytic sites. The present results support this interpretation, since the block copolymers exhibit distinctly higher conversions than the corresponding homopolymers, whereas the difference between the chloride and iodide analogs remains relatively small under the selected reaction conditions.

**Figure 5:**
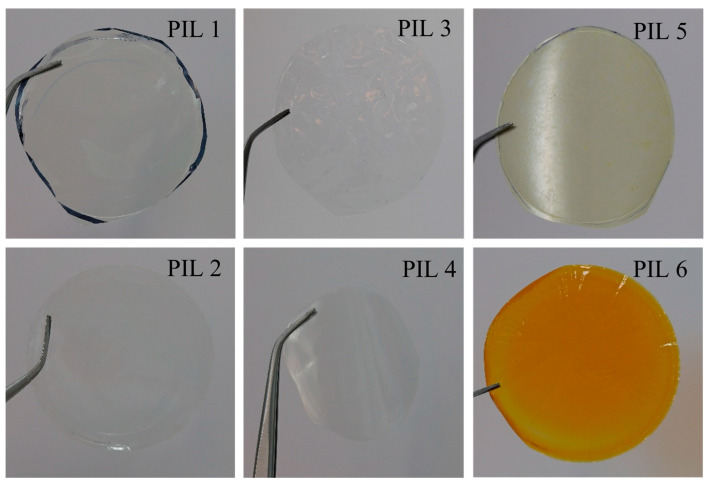
Photos of the PIL membranes.

For each PIL, a suitable organic solvent was selected that ensured complete dissolution of the polymer and an optimal evaporation rate. H_2_O has been found to be the best solvent for forming membranes from homopolymers. DMA is the best solvent for membranes from chloride block copolymers, and NMP is best for iodide block copolymers. Thus, membranes were formed from the following solutions: 15 wt. % PIL1 or PIL 2 / 85 wt. % H_2_O; 20 wt. % PIL 3 or PIL 4 / 80 wt. % DMA; 15 wt. % PIL 5 / 85 wt. % NMP; and 20 wt. % PIL 6 / 80 wt. % NMP. The thickness of the obtained films ranged from 50 to 95 μm. Photos of the membranes obtained from the synthesized PILs are shown in Figure 5.

**Figure 6:**
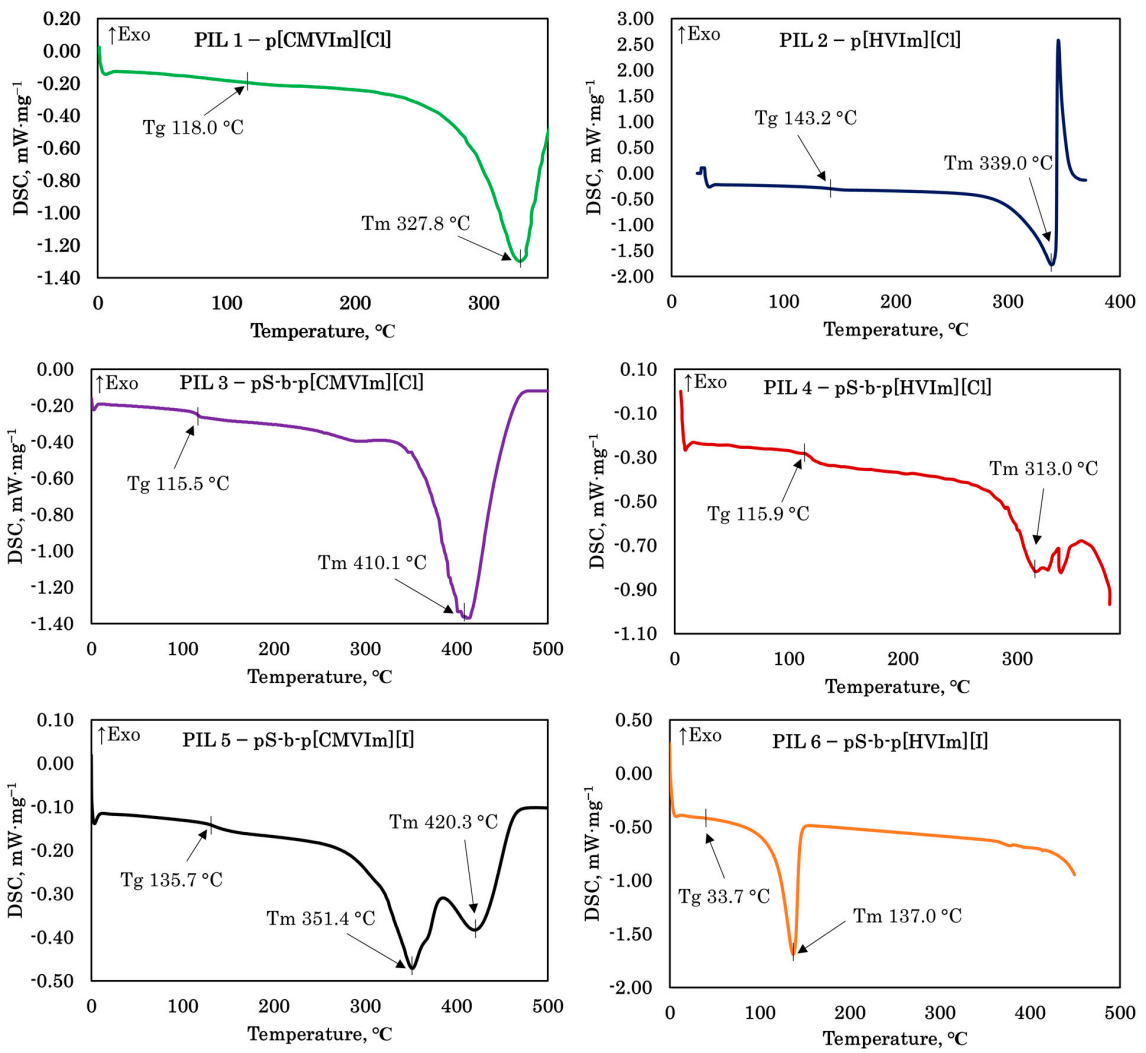
DSC thermograms of the synthesized PILs.

The DSC analysis revealed pronounced differences in the thermal behavior of the synthesized PILs depending on both the polymer architecture and the counter-anion (Figure 6). The homopolymers PIL 1 (p[CMVIm][Cl]) and PIL 2 (p[HVIm][Cl]) exhibited glass-transition temperatures of 118.0 °C and 143.2, respectively, indicating a more rigid chain environment in the hydroxyl-functionalized homopolymer, likely due to stronger interchain interactions. The chloride-containing block copolymers PIL 3 (pS-b-p[CMVIm][Cl]) and PIL 4 (pS-b-p[HVIm][Cl]) showed similar Tg values of 115.5 and 115.9 °C, suggesting comparable segmental mobility in the amorphous phase, while the high-temperature endothermic transitions observed at 410.1 and 313.0 °C, respectively, point to differences in the stability of ionic domains and supramolecular organization. A particularly strong effect was observed after anion exchange to iodide. For PIL 6 (pS-b-p[HVIm][I]), Tg decreased dramatically to 33.7 °C and an additional endothermic transition appeared at 137.0 °C, indicating pronounced plasticization of the ionic phase and the formation of less stable ordered domains. This interpretation is consistent with the SEM data, where iodide-containing samples, especially PIL 6, displayed the highest degree of microheterogeneity, interfacial defects, and internal delamination, as well as with their stronger pressure sensitivity in gas permeation experiments. In contrast, PIL 5 (pS-b-p[CMVIm][I]) retained a relatively high Tg of 135.7 °C and exhibited two high-temperature endothermic transitions at 351.4 and 420.3 °C, suggesting a more complex multiphase organization rather than simple softening.

**Figure 7:**
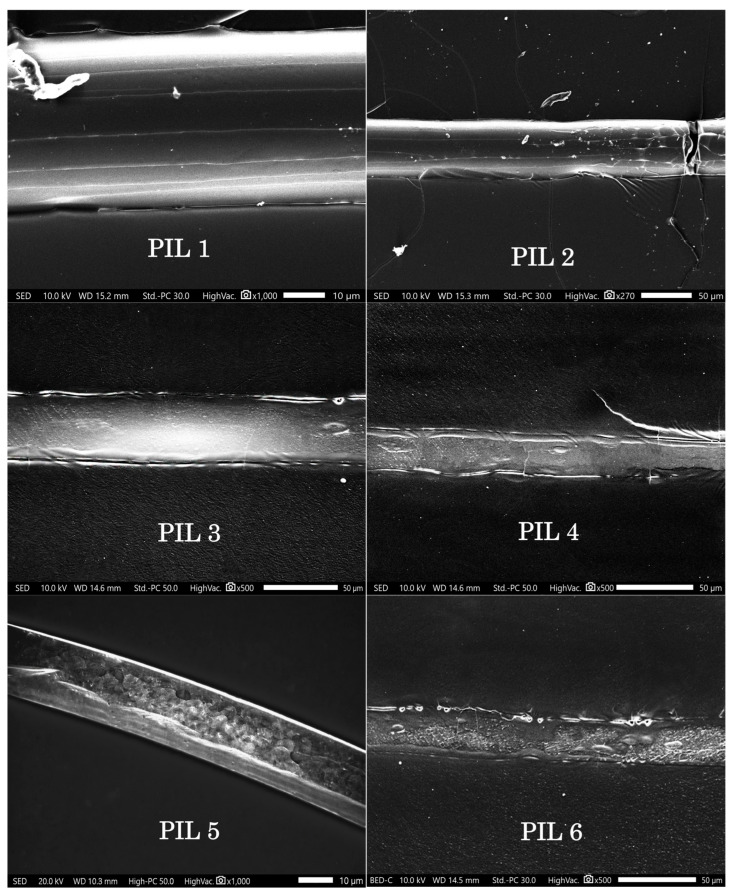
SEM micrographs of PIL membranes.

Analysis of the morphology of homo- and block copolymer samples was performed using a JSM-IT500 scanning electron microscope (SEM) from JEOL (Akishima, Japan). The presented SEM images of cross-sections of six membranes made of polymeric ionic liquids (PILs) and their block copolymers with polystyrene reveal significant differences in morphology associated with the chemical structure of the polymer and the nature of the anion (Figure 7). Samples PIL 1 (p[CMVim][Cl]) and PIL 2 (p[HVim][Cl]) are dense, homogeneous films without visible pores and defects, indicating a homogeneous, non-porous structure of the homopolymers. The introduction of a polystyrene block (PIL 3 pS-b-p[CMVim][Cl] and PIL 4 pS-b-p[HVim][Cl]) leads to microphase separation: the images reveal a nanostructured morphology with the possible presence of nanoscale channels, but macropores and cracks are absent, and the integrity of the membranes is preserved. Replacing the chloride anion with iodide (PIL 5 pS-b-p[CMVim][I] and PIL 6 pS-b-p[HVim][I]) radically alters the structure: domain coarsening, the appearance of micropores (ranging from tens to hundreds of nanometers in size), and defects in the form of voids at the interface with the epoxy resin and internal delaminations, particularly pronounced in sample PIL 6, are observed. Thus, the analysis shows that dense, defect-free structures are formed in chloride-containing materials, whereas the bulky I^−^ anion increases free volume and reduces mechanical stability, leading to heterogeneity and defects. Samples PIL 1–PIL 4 are the most homogeneous and suitable for creating selective dense membranes, while the iodide analogs require further optimization to eliminate structural imperfections.

**Figure 8:**
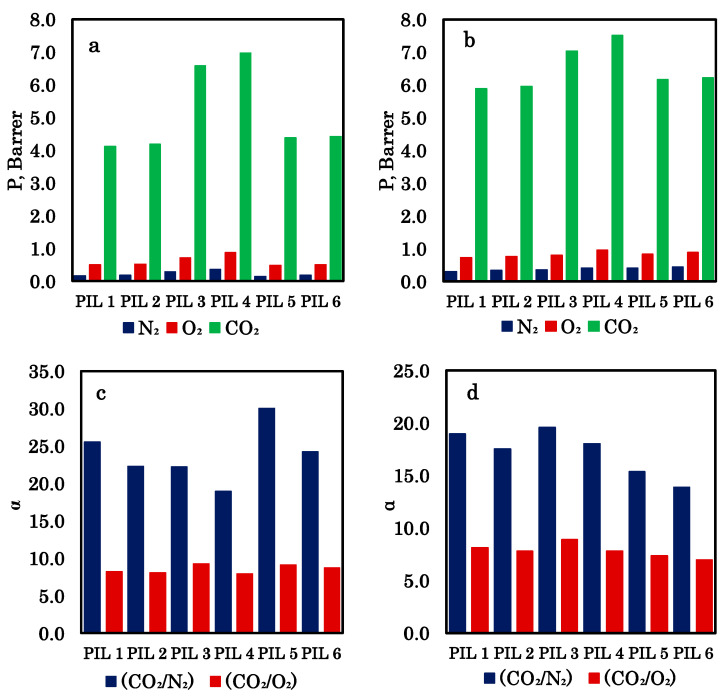
Mass transfer properties of PIL membranes for pure gases: permeability coefficients at 0.1 MPa (**a**) and 0.5 MPa (**b**); selectivity at 0.1 MPa (**c**) and 0.5 MPa (**d**).

The results of determining the mass transfer properties of PIL membranes for pure gases, carried out at two pressures (0.1 and 0.5 MPa), are presented in Figure 8 and Table 1. The homopolymers p[CMVIm][Cl] and p[HVIm][Cl] show the lowest CO_2_ permeability values (4.1–6.0 Barrer) among all the materials tested. At the same time, they exhibit high ideal selectivity for CO_2_/N_2_ (up to 25.5) and CO_2_/O_2_ (around 8). As pressure increases, the permeability of all gases rises, while selectivity decreases slightly. This behavior is typical of glassy polymers, where CO_2_-induced plasticization may occur. The two homopolymers have similar properties, although p[HVIm][Cl] demonstrates slightly higher permeability at the cost of marginally lower selectivity.

Introducing a polystyrene block into the structure (block copolymers pS-b-p[CMVIm][Cl] and pS-b-p[HVIm][Cl]) leads to a significant increase in CO_2_ permeability (6.6–7.5 Barrer) compared to the homopolymers. This enhancement is attributed to microphase separation and the formation of continuous transport channels within the ionic domains. The CO_2_/N_2_ selectivity remains high (18–22), though it is somewhat lower than that of the homopolymers. The highest CO_2_ permeability is observed for pS-b-p[HVIm][Cl] (6.96–7.51 Barrer), while the best CO_2_/O_2_ selectivity belongs to pS-b-p[CMVIm][Cl] (9.22–8.91). Thus, block copolymers with chloride anions appear most promising for achieving high CO_2_ flux while maintaining reasonable selectivity.

At low pressure, the CO_2_ permeability of iodide-containing samples (4.38–4.42 Barrer) is close to that of the homopolymers and noticeably lower than that of their chloride counterparts. However, in this case, the CO_2_/N_2_ selectivity reaches its maximum values (30.0 for CMVIm and 24.2 for HVIm), surpassing all other membranes. As pressure increases, CO_2_ permeability rises sharply (up to 6.17–6.23 Barrer), almost reaching the level of chloride block copolymers. At the same time, CO_2_/N_2_ selectivity drops to 15.4 and 13.9, respectively, becoming the lowest among all samples. This behavior can be explained by stronger plasticization of iodide-containing membranes under high-pressure CO_2_: the larger iodide ion likely creates greater free volume, which promotes the diffusion of all gases at elevated pressure but reduces the ability for selective separation.

Iodide block copolymers are the most pressure-sensitive materials. Their CO_2_ permeability shows the most pronounced increase with pressure (by a factor of 1.4–1.5), and their selectivity loss is the largest. In contrast, chloride block copolymers and homopolymers exhibit moderate permeability growth (by a factor of 1.1–1.2) and only a slight decrease in selectivity.

**Figure 9:**
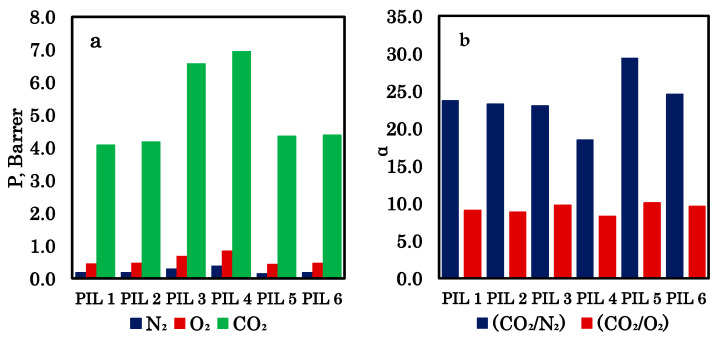
Mass transfer properties of PIL membranes for mixed gases at 0.15 MPa: permeability coefficients (**a**) and selectivity (**b**).

The mass transfer properties of PIL membranes studied for mixed gas, which imitates dry flue gas, are given in Figure 9 and Table 2. The homopolymers p[CMVIm][Cl] and p[HVIm][Cl] showed CO_2_ permeability values of 4.08 and 4.16 Barrer, respectively, which are very close to those obtained in single-gas measurements. Their CO_2_/N_2_ selectivities were around 23.3–23.7, slightly lower than the ideal selectivities but still quite high. The homopolymers performed similarly, with p[HVIm][Cl] giving marginally higher permeability at the expense of a small reduction in selectivity.

Introducing a polystyrene block led to a significant increase in CO_2_ permeability. The block copolymer pS-b-p[CMVIm][Cl] exhibited a CO_2_ permeability of 6.56 Barrer, which matched its single-gas value, along with a CO_2_/N_2_ selectivity of 23.0 and a CO_2_/O_2_ selectivity of 9.74. In contrast, pS-b-p[HVIm][Cl] showed the highest CO_2_ permeability among all samples at 6.94 Barrer, but its selectivities dropped to 18.45 for CO_2_/N_2_ and 8.27 for CO_2_/O_2_. This decline was more pronounced than in single-gas tests, suggesting that the hydroxyethyl-containing copolymer is more affected by competitive sorption or plasticization in the gas mixture.

The iodide-containing block copolymers displayed a different behavior. Both pS-b-p[CMVIm][I] and pS-b-p[HVIm][I] had CO_2_ permeabilities around 4.34–4.38 Barrer, similar to the homopolymers and noticeably lower than their chloride counterparts. However, their selectivities reached the highest values observed: 29.37 for CO_2_/N_2_ and 10.05 for CO_2_/O_2_ in the case of the CMVIm-based sample, and 24.53 and 9.58 for the HVIm-based one. These selectivities were even slightly better than those recorded in single-gas experiments, indicating that the larger iodide ion creates a free-volume environment that favors CO_2_ transport while effectively hindering nitrogen and oxygen in the mixture.

When comparing the mixed-gas and single-gas results, CO_2_ permeability values were remarkably consistent across all polymers, with differences typically within 0.1 Barrer. This suggests that competitive sorption does not significantly reduce the CO_2_ flux. Selectivity trends also followed the single-gas patterns, although the iodide samples showed a slight improvement in CO_2_/O_2_ selectivity under mixed-gas conditions. Overall, the block copolymers with chloride anions offered the best combination of high CO_2_ permeability and reasonable selectivity, while the iodide-containing materials excelled in selectivity, making them attractive for applications where separation purity is critical. The mixed-gas experiments confirmed that these membranes retain their key transport characteristics in more realistic feed conditions.

The CO_2_ permeability/selectivity combination obtained for the present membranes is comparable to that reported for a number of dense PIL-based membranes (PILMs) and supported ionic liquid membranes (SILMs) in the literature. In particular, previously reported imidazolium-containing PIL membranes showed CO_2_ permeability values in the approximate range of 4.1–9.2 Barrer, with CO_2_/N_2_ selectivity typically between 32 and 40, whereas some other PIL membranes exhibited even lower CO_2_ permeability of about 0.9–1.0 Barrer with CO_2_/N_2_ selectivity of 15.3–19.1 [17,27]. A comparison of the mass transport properties of membranes is given in Table 3. Although more highly optimized crosslinked PIL and PIL/IL composite membranes may achieve substantially higher CO_2_ permeability, these materials were designed exclusively for gas separation [47]. By contrast, the present membranes are bifunctional materials combining gas separation with catalytic CO_2_ conversion, and thus, their significance lies in the integration of these two functions within a single material platform.

The analysis of the experimentally obtained diffusion coefficients and calculated sorption coefficients shows that the permeability of the studied PIL membranes is governed by the combined contribution of both diffusion and sorption, in accordance with the solution–diffusion model. At the same time, the gas selectivity is determined mainly by the sorption term, since the diffusion coefficients of N_2_, O_2_, and CO_2_ are of the same order of magnitude, whereas the sorption coefficients of CO_2_ are significantly higher than those of the other gases for all membranes. Thus, the preferential transport of CO_2_ is primarily associated with its stronger affinity toward the ionic domains of the PIL matrix. At the same time, upon transition from the homopolymers to the chloride-containing block copolymers and then to the iodide-containing block copolymers, the diffusional contribution becomes more pronounced. This trend can be explained by the gradual change in membrane structure from dense and homogeneous in the homopolymers to microphase-separated in the chloride-containing block copolymers and further to more heterogeneous, free-volume-rich structures in the iodide-containing block copolymers. As a result, gas diffusion becomes progressively less restricted, whereas the sorption contribution, especially for CO_2_, decreases. Therefore, the absolute permeability is controlled by both transport factors, while the differences in selectivity are governed mainly by the much higher sorption of CO_2_.

## 4. Conclusions

In this study, a family of polymeric ionic liquids based on imidazolium cations with chloride or iodide counterions, both as homopolymers and as block copolymers with polystyrene, was successfully synthesized and evaluated as bifunctional materials for combined CO_2_ separation and catalytic conversion. The materials’ catalytic performance during the cycloaddition of CO_2_ to epichlorohydrin revealed that the incorporation of a polystyrene block significantly enhances the conversion, reaching values above 80% for all block copolymers compared to 68–77% for the homopolymers, while selectivity remained high. The anion effect was less pronounced under the reaction conditions, indicating that the polymer architecture plays a dominant role in determining catalytic efficiency. Gas transport measurements for pure gases and a simulated flue gas mixture showed that the chloride-containing block copolymers offer the best balance between CO_2_ permeability and CO_2_/N_2_ selectivity, making them attractive for applications where both flux and separation efficiency are required. In contrast, the iodide-containing block copolymers exhibited high CO_2_/N_2_ selectivity under mixed-gas conditions, reaching values up to 29.4, but suffered from stronger pressure-induced plasticization and a more pronounced loss of selectivity at elevated pressures. Scanning electron microscopy analysis confirmed that chloride-containing materials form dense, defect-free films, while the more voluminous iodide anion leads to increased free volume, microphase separation, and structural heterogeneity, which correlates well with the gas transport characteristics of these membranes. Overall, these results demonstrate that by carefully selecting the polymer architecture (homopolymer or block copolymer) and the anion, it is possible to tailor the properties of PIL-based membranes to either maximize CO_2_ flux or prioritize separation selectivity. The ability to combine catalytic activity with efficient CO_2_ transfer within a single material platform represents a significant step toward the development of integrated membrane reactors for carbon capture and conversion. This potentially eliminates the need for additional units, thereby reducing the energy and cost losses associated with existing technologies.

## Figures and Tables

**Table 1:** Mass transfer properties of PIL membranes for pure gases.

№	P, Barrer			D × 10^8^, cm^2^ s^−1^			S × 10^3^, cm^3^ cm^−3^ cmHg^−1^			α	
	N_2_	O_2_	CO_2_	N_2_	O_2_	CO_2_	N_2_	O_2_	CO_2_	CO_2_/N_2_	CO_2_/O_2_
PIL 1	0.16 ^a^	0.50 ^a^	4.11 ^a^	5.62 ^a^	7.14 ^a^	1.05 ^a^	0.28 ^a^	0.70 ^a^	39.22 ^a^	25.54 ^a^	8.24 ^a^
	0.31 ^b^	0.73 ^b^	5.89 ^b^							18.95 ^b^	8.10 ^b^
PIL 2	0.19 ^a^	0.52 ^a^	4.19 ^a^	5.63 ^a^	7.75 ^a^	1.34 ^a^	0.34 ^a^	0.67 ^a^	31.16 ^a^	22.30 ^a^	8.08 ^a^
	0.34 ^b^	0.77 ^b^	5.96 ^b^							17.55 ^b^	7.79 ^b^
PIL 3	0.30 ^a^	0.71 ^a^	6.58 ^a^	5.61 ^a^	8.14 ^a^	2.32 ^a^	0.53 ^a^	0.87 ^a^	28.33 ^a^	22.22 ^a^	9.22 ^a^
	0.36 ^b^	0.79 ^b^	7.04 ^b^							19.60 ^b^	8.91 ^b^
PIL 4	0.37 ^a^	0.88 ^a^	6.96 ^a^	6.41 ^a^	8.52 ^a^	2.55 ^a^	0.58 ^a^	1.03 ^a^	27.28 ^a^	18.96 ^a^	7.90 ^a^
	0.42 ^b^	0.96 ^b^	7.51 ^b^							18.02 ^b^	7.80 ^b^
PIL 5	0.15 ^a^	0.48 ^a^	4.38 ^a^	5.10 ^a^	7.02 ^a^	2.93 ^a^	0.29 ^a^	0.68 ^a^	14.94 ^a^	30.03 ^a^	9.10 ^a^
	0.40 ^b^	0.84 ^b^	6.17 ^b^							15.39 ^b^	7.33 ^b^
PIL 6	0.18 ^a^	0.51 ^a^	4.42 ^a^	4.98 ^a^	7.11 ^a^	3.76 ^a^	0.36 ^a^	0.72 ^a^	11.74 ^a^	24.19 ^a^	8.67 ^a^
	0.45 ^b^	0.89 ^b^	6.23 ^b^							13.90 ^b^	6.98 ^b^

0.1 MPa (^a^); 0.5 MPa (^b^); 25 °C; 1 Barrer = 10^−10^cm^3^ (STP) cm cm^−2^ s^−1^ cmHg^−1^.

**Table 2:** Mass transfer properties of PIL membranes for mixed gases.

№	P, Barrer			D × 10^8^, cm^2^ s^−1^			S × 10^3^, cm^3^ cm^−3^ cmHg^−1^			α	
	N_2_	O_2_	CO_2_	N_2_	O_2_	CO_2_	N_2_	O_2_	CO_2_	CO_2_/N_2_	CO_2_/O_2_
PIL 1	0.17	0.45	4.08	5.63	7.11	1.04	0.30	0.63	39.11	23.68	9.05
PIL 2	0.18	0.47	4.16	5.67	7.73	1.35	0.32	0.61	30.91	23.26	8.85
PIL 3	0.29	0.67	6.56	5.56	8.13	2.36	0.52	0.82	27.83	22.99	9.74
PIL 4	0.38	0.84	6.94	6.49	8.5	2.57	0.59	0.99	26.99	18.45	8.27
PIL 5	0.15	0.43	4.34	5.21	6.99	3.04	0.29	0.62	14.29	29.37	10.05
PIL 6	0.18	0.46	4.38	4.97	7.1	3.91	0.36	0.65	11.21	24.53	9.58

0.15 MPa and 25 °C; 1 Barrer = 10^−10^cm^3^ (STP) cm cm^−2^ s^−1^ cmHg^−1^.

**Table 3:** Comparison of mass transport properties of membranes developed in this work with PILMs or SILMs from the literature.

Membrane	P, Barrer		α	
	N_2_	CO_2_	CO_2_/N_2_	
PIL 1	0.16	4.11	25.54	This work ^a^
PIL 2	0.19	4.19	22.30	
PIL 3	0.30	6.58	22.22	
PIL 4	0.37	6.96	18.96	
PIL 5	0.15	4.38	30.03	
PIL 6	0.18	4.42	24.19	
PIL pyrrolidinium–10IL	0.40	11.5	28.75	[27] ^b^
PIL imidazolium–10IL	0.63	14.90	23.70	
PIL pyridinium–10IL	1.01	20.40	20.10	
PIL ammonium–10IL	0.37	9.44	25.80	
PIL cholinium–10IL	0.11	3.66	34.70	
pVBmimBF_4_	0.8	5.0	5.88	[17] ^c^
pVBmimPF_6_	0.2	3.0	12.0	
pVBmimTf_2_N	0.9	17.0	19.1	
pVBPyBF_4_	0.8	6.0	7.6	
pVBPyPF_6_	0.3	2.0	6.6	
pVBPyTf_2_N	1.0	15.0	15.3	

^a^ 0.15 MPa and 25 °C; ^b^ 0.1 MPa and 20 °C; ^c^ 0.13 MPa and 20 °C; 1 Barrer = 10^−10^cm^3^ (STP) cm cm^−2^ s^−1^ cmHg^−1^.

## Data Availability

The original contributions presented in this study are included in the article. Further inquiries can be directed to the corresponding author.
